# Supplementary material for: Association between oral microbiome and seven types of cancers in East Asian population: a two-sample Mendelian randomization analysis
Source: Front Mol Biosci. 2023 Nov 21;10:1327893. doi: 10.3389/fmolb.2023.1327893 (PMC10702768; doi:10.3389/fmolb.2023.1327893)
Supplement: Supplementary file 1 [file DataSheet1.ZIP › Supplementary Materials/MR plots of saliva/saliva═╝/Prostate cancer/pheno.2050_to_prostate cancer_funnel.pdf]

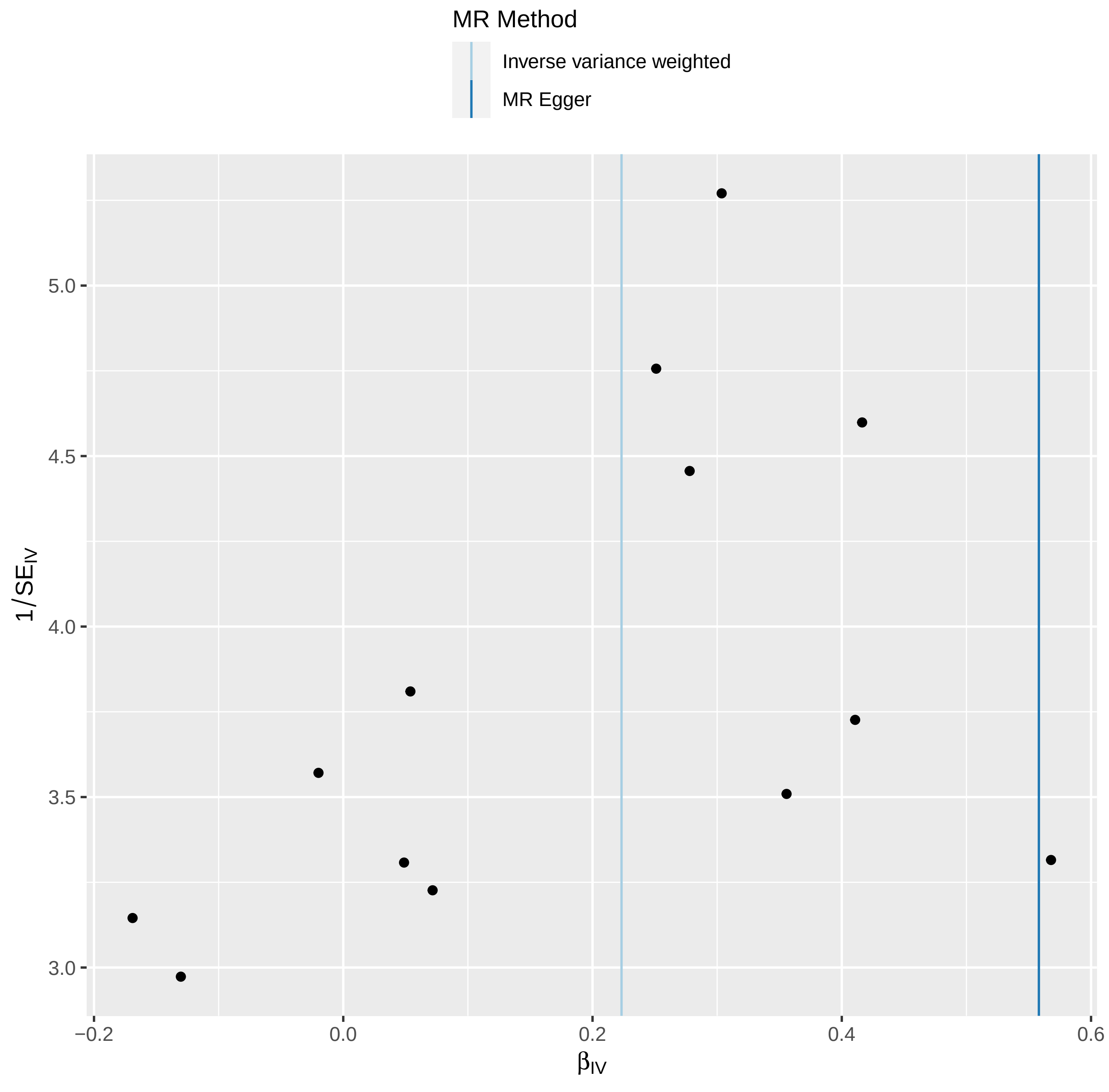
MR Method
Inverse variance weighted
MR Egger
5.0
4.5
4.0
3.5
3.0
$1/SE_{IV}$
−0.2
0.0
0.2
0.4
0.6
β_{IV}
